# Stress pulmonary circulation parameters assessed by a cardiovascular magnetic resonance in patients after a heart transplant

**DOI:** 10.1038/s41598-022-09739-z

**Published:** 2022-04-12

**Authors:** Lukáš Opatřil, Roman Panovsky, Mary Mojica-Pisciotti, Jan Máchal, Jan Krejčí, Tomáš Holeček, Lucia Masárová, Věra Feitová, Július Godava, Vladimír Kincl, Tomáš Kepák, Gabriela Závodná, Lenka Špinarová

**Affiliations:** 1 First Department of Internal Medicine and Cardioangiology, St. Anne’s University Hospital, Pekarska 53, 656 91 Brno, Czech Republic; 2 International Clinical Research Centre, St. Anne’s University Hospital, Brno, Czech Republic; 3 Faculty of Medicine, Masaryk University, Brno, Czech Republic; 4 First Department of Internal Medicine and Cardioangiology, International Clinical Research Centre, Faculty of Medicine, Masaryk University, St. Anne’s University Hospital, Brno, Czech Republic; 5 Department of Pathophysiology, Faculty of Medicine, Masaryk University, Brno, Czech Republic; 6 Department of Medical Imaging, St. Anne’s University Hospital, Brno, Czech Republic; 7 Department of Paediatric Oncology, University Hospital Brno, Brno, Czech Republic

**Keywords:** Cardiology, Diagnostic markers, Cardiovascular diseases

## Abstract

Rest pulmonary circulation parameters such as pulmonary transit time (PTT), heart rate corrected PTT (PTTc) and pulmonary transit beats (PTB) can be evaluated using several methods, including the first-pass perfusion from cardiovascular magnetic resonance. As previously published, up to 58% of patients after HTx have diastolic dysfunction detectable only in stress conditions. By using adenosine stress perfusion images, stress analogues of the mentioned parameters can be assessed. By dividing stress to rest biomarkers, potential new ratio parameters (PTT ratio and PTTc ratio) can be obtained. The objectives were to (1) provide more evidence about stress pulmonary circulation biomarkers, (2) present stress to rest ratio parameters, and (3) assess these biomarkers in patients with presumed diastolic dysfunction after heart transplant (HTx) and in childhood cancer survivors (CCS) without any signs of diastolic dysfunction. In this retrospective study, 48 patients after HTx, divided into subgroups based on echocardiographic signs of diastolic dysfunction (41 without, 7 with) and 39 CCS were enrolled. PTT was defined as the difference between the onset time of the signal intensity increase in the left and the right ventricle. PTT in rest conditions were without significant differences when comparing the CCS and HTx subgroup without diastolic dysfunction (4.96 ± 0.93 s vs. 5.51 ± 1.14 s, p = 0.063) or with diastolic dysfunction (4.96 ± 0.93 s vs. 6.04 ± 1.13 s, p = 0.13). However, in stress conditions, both PTT and PTTc were significantly lower in the CCS group than in the HTx subgroups, (PTT: 3.76 ± 0.78 s vs. 4.82 ± 1.03 s, p < 0.001; 5.52 ± 1.56 s, p = 0.002). PTT ratio and PTTc ratio were below 1 in all groups. In conclusion, stress pulmonary circulation parameters obtained from CMR showed prolonged PTT and PTTc in HTx groups compared to CCS, which corresponds with the presumption of underlying diastolic dysfunction. The ratio parameters were less than 1.

## Introduction

Pulmonary circulation biomarkers obtained with non-invasive methods are not new themselves. Acquired by different modalities, including radionuclide imaging^1^, contrast-enhanced transthoracic or transesophageal echocardiography^2,3^, or computed tomography^4^, they have been studied for decades.

One of the latest techniques to assess pulmonary circulation parameters is magnetic resonance imaging (MRI). Firstly, through MRI angiography^5^, and later on by the first-pass perfusion from cardiovascular magnetic resonance (CMR)^6–8^. The latter option offers advantages such as using an already employed sequence and opens new opportunities with retrospective studies, although data are still limited.

From the analysis of rest perfusion images, biomarkers such as pulmonary transit time (PTT), pulmonary transit beats (PTB) and pulmonary blood volume index (PBVI), can be obtained. Likewise, by using adenosine stress perfusion images, their analogues, the stress pulmonary transit time (PTT_S) and the stress pulmonary transit beats (PTB_S), can also be determined. To our knowledge, so far, just one article reported stress parameters, but only in hypertrophic cardiomyopathy (HCM)^7^.

PTT has already shown to be increased in both heart failure with reduced and preserved ejection fraction^6,9^. As previously published by Meluzin et al.^10^, by pulmonary capillary wedge pressure acquired by right heart catheterization, 16% of patients after heart transplant (HTx) showed signs of diastolic dysfunction in rest condition, but in stress condition, another 58% showed these signs^10^. Therefore, we hypothesise that in a population after HTx, the stress biomarkers will be prolonged compared to a group of patients without any signs of systolic or diastolic function impairment.

We further assume that in a healthy population, the stress pulmonary transit biomarkers should be shorter and assessing not only the absolute values of rest and stress parameters, but also their corresponding ratio could be valuable.

As far as we know, these biomarkers have not been studied before on patients after HTx or in the childhood cancer survivors (CCS).

This study aimed to (1) provide more evidence about stress pulmonary circulation biomarkers, (2) present and investigate stress to rest ratio parameters, and (3) assess these biomarkers in patients with presumed diastolic dysfunction after heart transplant (HTx) and in CCS without diastolic dysfunction.

## Methods

### Study design and population

This retrospective study was performed in accordance with the Declaration of Helsinki (2000) of the World Medical Association. The Ethics Committee of the Faculty of Medicine, Masaryk University, confirmed that this study has been performed on data of patients already participating in other research studies with signed Informed Consent and therefore, according to the Czech legislation, no specific new approval of the ethics committee was required.

In this retrospective study, 48 subjects after HTx and 39 CCS patients were included. Detailed inclusion and exclusion criteria are shown in Table 1.

**Table 1:** Inclusion and exclusion criteria.

Inclusion criteria		Exclusion criteria	
HTx	CCS	HTx	CCS
1 year ± 30 days after HTx	Adults after cardiotoxic chemotherapy in childhood	sPAP > 40 mmHg	sPAP > 40 mmHg
Stress CMR perfusion available	Stress CMR perfusion available	–	Any signs of systolic or diastolic impairment
Echocardiographic examination including E/E’, E/A measurement and pulmonary systolic artery pressure assessment	Echocardiographic examination including E/E’, E/A measurement and pulmonary systolic artery pressure assessment		-
≤ 30 days between CMR and echocardiography	≤ 30 days between CMR and echocardiography		

*CMR* cardiovascular magnetic resonance, *CCS* childhood cancer survivors, *HTx* heart transplant group, *sPAP* systolic pulmonary artery pressure.

The HTx group consisted of patients who underwent HTx for different diagnoses and were being monitored at our center. In the first year after transplantation, a detailed follow-up, including myocardial biopsies and other examinations, took place. During the first-year check-up, they underwent more thorough examinations with CMR, including a stress perfusion test.

The CCS group were patients after cardiotoxic chemotherapy in young or adolescent age treated at the paediatric oncology. Patients were treated with anthracyclines in 74.3%, the rest with Cisplatin and high dose Carboplatin. In terms of specific drugs, 93% of patients treated with anthracyclines received Doxorubicin. All subjects were examined and monitored at our department between the years 2016 and 2020. Only patients that after a thorough examination including transthoracic echocardiography (TTE) and CMR methods showed no signs of systolic or diastolic function impairment or another cardiac pathology were enrolled.

Inclusion criteria required a CMR examination with contrast methods and stress perfusion and TTE without signs of pulmonary hypertension (PH) within 30 days of CMR. Stress perfusions were indicated to exclude coronary artery disease, since it was already detected in some patients treated with anthracyclines or alkylating agents^11,12^ and were performed as part of the study.

Patients in the HTx group were divided into subgroups based on the presence of diastolic dysfunction signs from TTE.

### Transthoracic echocardiography

All patients underwent the standard TTE exam, and all examinations were performed by experienced cardiologists in our center and according to the established guidelines.

Signs of PH were defined in accordance with 2015 guidelines^13^ as estimated pulmonary systolic artery pressure above 40 mmHg, calculated from tricuspid regurgitation jet velocity. The left ventricular diastolic dysfunction was defined as the presence of a grade II or higher diastolic dysfunction assessed by Doppler echocardiography of transmitral velocities of transmitral flow and tissue Doppler imaging of mitral annular velocity.

### CMR protocol

The CMR studies were performed similarly as in the case of previous articles from our workplace^14^—with the standard protocol using 1.5 T scanners (Ingenia, Philips Medical Systems, Best, The Netherlands) equipped with 5- and 32-element phased-array receiver coils allowing for the use of parallel acquisition techniques in the supine position in repeated breath-hold. Functional imaging using balanced steady-state free precession (SSFP, b-TFE) cine sequences included four-chamber, two-chamber and left ventricular outflow tract (LVOT) long-axis views, and a short-axis (SAX) stack from the cardiac base to the apex in the plane perpendicular to the LV long axis. Wall motion abnormalities were visually assessed. LV functional and morphological parameters were calculated from the SAX stack using the summation-of-disc methods following the recommendations on post-processing evaluation from the Society for Cardiovascular Magnetic Resonance^15^.

Late gadolinium enhancement (LGE) images in all long-axis views and the SAX stack were acquired 10 min after an intravenous bolus of a total 0.2 mmol/kg (stress and rest dual-boluses for perfusion and the bolus of remaining contrast injected after the rest perfusion) of the gadolinium-based contrast agent gadobutrol (Gadovist, Bayer-Schering Pharma, Germany) using inversion-recovery turbo field echo sequence (IR-TFE) and, in case of doubt, also by phase-sensitive inversion recovery (PSIR) TFE. Both 2-dimensional and 3-dimensional data acquisitions were performed. LGE was defined as an area of visually identified contrast enhancement higher than the mean signal intensity of an adjacent area of the reference myocardium.

### CMR stress perfusion

CMR first‐pass contrast‐enhanced myocardial perfusion images were acquired by a b-TFE sequence in three SAX sections (basilar, midventricular, and apical) with these parameters: field of view 300 × 300 mm, reconstruction matrix 224, slice thickness 10 mm, acquisition voxel size 2.5 × 2.5 mm, time to repetition (TR) ≈ 2.2 ms, echo time (TE) ≈ 1.1 ms, flip angle 50°, SENSE factor 2.3, number of dynamics = 90, non-shared saturation prepulse. Stress perfusion was acquired after a 4 min adenosine infusion at 140 μg/kg/min. Rest perfusion was acquired 10 min after the stress perfusion with the same parameters. MR perfusion was performed during a dual-bolus administration of gadolinium-based contrast agent gadobutrol. The dual-bolus protocol was adapted from Ishida et al.^16^. A volume and rate of 0.005 mmol/kg and 4 ml/s for the pre-bolus and 0.05 mmol/kg and 4 ml/s for the main-bolus were used. Stress and rest volumes and rates were matched.

### Pulmonary circulation biomarkers analyses

**Figure 1:**
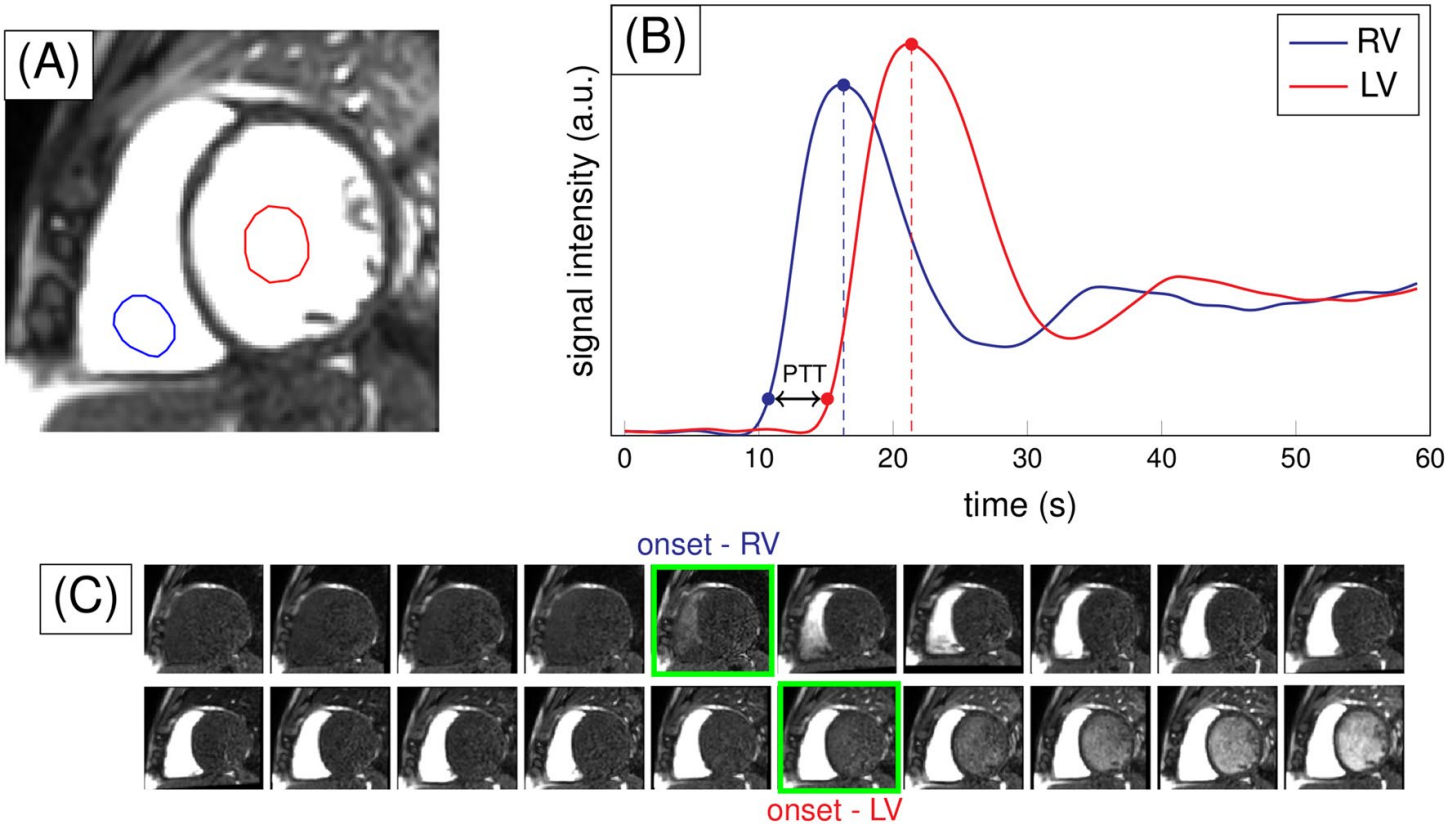
Schematics of the calculation of pulmonary transit time (PTT). (**A**) Region of interests manually traced in the right ventricle (RV, blue) and the left ventricle (LV, red). (**B**) Simulated data of the signal intensity (SI) vs time for the RV and LV. The maxima of the SI are marked. The PTT is the difference between the onsets, determined as the signal at 10% of the maximum values. (**C**) Example of the detection of the onset points in the corrected images. Only a fraction of the images is shown.

The PTT values were estimated from SAX rest and stress first-pass perfusion images, using an in-house developed algorithm implemented in MATLAB^®^ 9.8 (R2020a) (The Mathworks Inc., Natick, MA, USA). The images were acquired without breath-hold. The analysis starts with applying a motion correction algorithm to optimize imaging registration and avoid potential contamination from pixels in the blood pool. The algorithm initially performs a rigid transformation to improve the misalignment present in the images and ease the following registration steps. Subsequently, an affine transformation, optimized according to the One Plus One Evolutionary strategy^17^, is applied. The method generates a modification in the image using a current transformation (parent) and a newer one (descendant). Then, it estimates the similarity value, and according to it, keeps the parent or the descendant for a new evolutionary step. Finally, the algorithm uses mutual information as a similarity criterion^18^. The registration accuracy is visually assessed. Regions of interest (ROIs) in the right ventricle (RV) and the LV are manually traced in both the rest and stress corrected images for the mid-ventricular slice and, in case of repetitive misregistration, in the basal one. The ROIs automatically propagate throughout the stack of images, their average is computed in each one, and signal intensity (SI) curves vs time are obtained. The algorithm recognizes the maximum SI value in each case and determines a threshold (5–10% of the maximum) for establishing the onset SI. The measurement was done considering the main bolus of the dual bolus technique and confirmed with the pre-bolus. PTT was defined as the difference between the onset time in the LV and the RV, and PTB is the number of frames between these times (see Fig. 1). The PBVI, in ml/m^2^, was calculated as the product of the PTB and the RV stroke volume (RV SV), divided by the body surface area^8^.

The pulmonary transit time ratio (PTT ratio) and pulmonary transit time ratio with heart rate (HR) correction (PTTc ratio) were calculated.

Where applicable, Bazett's formula (^19^), commonly used to standardize the QT interval across different heart rates, was used to correct the values for HR as previously reported by Ricci et al.^7^. The formula considers a PTT or PTT_S at a given heart rate and normalizes it by the square root of the corresponding RR interval per second, i.e., ⎷(RR interval/1 s)^19^. Therefore, the corrected parameters are PTTc (s) = PTT (s)/⎷(RR interval/1 s) and PTTc_S (s) = PTT_S (s)/⎷ (RR interval/1 s). Ratio parameters (PTT ratio and PTTc ratio) were calculated as stress parameters divided by rest parameters; therefore, PTT ratio = PTT_S/ PTT and PTTc ratio = PTTc_S/ PTTc.

### Statistical analysis

The categorical data were compared using the χ^2^ test. In case the null hypothesis was refuted, a series of three Fisher exact tests followed by Bonferroni correction was employed. The continuous data generally followed the Gaussian distribution, as assessed by the Kolmogorov–Smirnov test and visual inspection of histograms. In the case of R-R intervals, square root transformation was applied. One-way ANOVA was used to compare the groups, followed by Tukey Post-hoc test for unequal N. Discrete data (PTB, PTB_S) were assessed using the Kruskal–Wallis test and the Dunn Post-hoc test. In all cases, α = 0.05 was used to define a statistically significant result.

## Results

### Patient’s population

Basic clinical, TTE and CMR group characteristics are summarized in Table 2.

**Table 2:** Baseline clinical, TTE and CMR parameters.

	CCS	HTx_A	HTx_B	HTx	p (CCS vs. HTx_A)	p (CCS vs. HTx_B)	p (HT_A vs. HTx_B)	p (CCS vs. HTx)
**Clinical parameters**								
Number of patients	39	41	7	48	NA	NA	NA	NA
Age (years)	24. 7 ± 4.6	51.4 ± 12	51.2 ± 14	51.4 ± 12.1	** < 0.001**	** < 0.001**	> 0.99	** < 0.001**
Height	172.7 ± 7.3	175.6 ± 8.9	172.7 ± 10.3	175.2 ± 9.2	0.31	> 0.99	0.8	0.15
Weight (kg)	68.1 ± 14	84.5 ± 15.5	89.6 ± 13.8	85.2 ± 15.5	** < 0.001**	**0.02**	0.79	** < 0.001**
Gender (male), n (%)	30 (77)	34 (83)	6 (86)	40 (83)	0.60	> 0.99	> 0.99	0.59
BMI (kg/m^2^)	22.8 ± 4.3	27.3 ± 4.1	30.0 ± 3.8	27.7 ± 4.1	** < 0.001**	**0.004**	0.44	** < 0.001**
LVEF (%)	62.7 ± 5.8	62.7 ± 4.8	59.7 ± 6.6	62.3 ± 5.1	0.91	0.47	0.57	0.41
E/A ratio	1.56 ± 0.46	1.62 ± 0.4	1.92 ± 0.79	1.66 ± 0.48	0.98	0.48	0.51	0.31
E/E´ ratio	6.12 ± 1.19	8.7 ± 1.81	14 ± 7.9	9.58 ± 3.98	** < 0.001**	** < 0.001**	** < 0.001**	** < 0.001**
DD LV (mm)	42.03 ± 4.38	45.88 ± 4.92	45.29 ± 3.77	45.79 ± 4.74	** < 0.001**	0.37	0.97	** < 0.001**
**CMR parameters**								
LVEF (%)	60 ± 6	69.7 ± 6.7	59.6 ± 10.7	68.2 ± 8.1	** < 0.001**	0.99	**0.018**	** < 0.001**
LV SV (mL)	78.5 ± 18.5	68.1 ± 15.3	60 ± 14.3	66.9 ± 15.3	**0.021**	0.11	0.64	**0.002**
RV SV (%)	74.7 ± 16.9	70.3 ± 15.8	59.4 ± 15.4	68.7 ± 16.1	0.46	0.19	0.43	0.096
LV LGE, n (%)	0 (0)	5 (12)	2 (28.6)	7 (15)	NA	NA	NA	NA
Heart rate (bpm), rest	74.1 ± 13.4	82.2 ± 11.9	84.9 ± 9.8	82.1 ± 11.6	**0.016**	0.26	0.92	**0.002**
Heart rate (bpm), stress	103.5 ± 17.1	91.1 ± 10.1	91.1 ± 12.7	91.1 ± 10.3	** < 0.001**	0.22	> 0.99	** < 0.001**
Rest cardiac output (L/min)	5.33 ± 1.53	5.76 ± 1.24	6.36 ± 1.25	5.87 ± 1.27	0.65	0.48	0.72	0.23
Stress cardiac output (L/min)	11.62 ± 2.08	8.11 ± 1.64	8.22 ± 1.88	8.13 ± 1.87	**0.002**	0.53	0.91	** < 0.001**
Adenosine-induced perfusion defects, n (%)	0 (0)	2 (4.9)	0 (0)	2 (4.2)	NA	NA	NA	NA

Values are presented as mean ± SD unless otherwise indicated.

Statistically significant p values are in bold.

*BMI* body mass index, *LVEF* left ventricular ejection fraction, *LV SV* left ventricular stroke volume, *DD LV* left ventricular diastolic diameter, *RV SV* right ventricular systolic volume, *LV LGE* left ventricular late gadolinium enhancement, *TTE* transthoracic echocardiography, *CMR* cardiovascular magnetic resonance, *CCS* childhood cancer survivors, *HTx* heart transplant group, *HTx_A* heart transplant without diastolic dysfunction subgroup, *HTx_B* heart transplant with diastolic dysfunction subgroup.

In the CCS group, no patient showed signs of diastolic dysfunction. The most common indications for treatment were Hodgkin lymphoma (11 patients, 28%) followed by Non-Hodgkin lymphoma (9 patients, 23%) and acute lymphocytic leukaemia (7 patients, 18%).

In the HTx group, seven patients showed grade II or higher diastolic dysfunction. The HTx population was divided based on the presence of diastolic dysfunction as a subgroup without (HTx_A) and another with (HTx_B).

The most common indication for HTx was dilated cardiomyopathy (30 patients, 63%), followed by ischemic heart disease (7 patients, 15%).

Since the number of patients included in HTx_B subgroup was relatively low, we also compared the whole HTx group with the CCS.

### Pulmonary circulation biomarkers

The retrospective analysis of biomarkers was successful in all cases, and no adverse effects of adenosine application were observed.The CCS patients showed biomarker values as follows: PTT 4.96 ± 0.93 s, PTB 5.82 ± 0.91, PTT_S 3.76 ± 0.78 s, for the HTx group: PTT 5.59 ± 1.14 s, PTB 7.40 ± 1.65, PTT_S 4.92 ± 1.13 s, for the HTx_A population: PTT 5.51 ± 1.14 s, PTB 7.22 ± 1.62, PTT_S 4.82 ± 1.03 s and lastly for HTx_B population: PTT 6.04 ± 1.13 s, PTB 8.43 ± 1.51 and PTT_S 5.52 ± 1.56 s. Both PTTc and PTB in the CCS group were significantly lower than in the HTx and both HTx_A and the HTx_B subgroups. PTT showed significantly lower values only in the case of HTx and CCS group, when comparing to subgroups lower values without statistical significance were shown. In the case of stress parameters, both PTT_S and PTTc_S were significantly lower in the CCS group than in the HTx groups. The comparison between HTx_A and HTx_B shows trend toward lower values in the first group but without statistical significance. The PTT ratio was significantly lower in the CCS than in the HTx group and HTx_A subgroup.

In addition, PTT-derived parameters did not significantly correlate with cardiac output (CO) at rest, but correlated with the stress CO. There was a difference between the CCS and the HTx group and HTx_A subgroup in the values of stress CO. There was no difference in the resting values.

**Figure 2:**
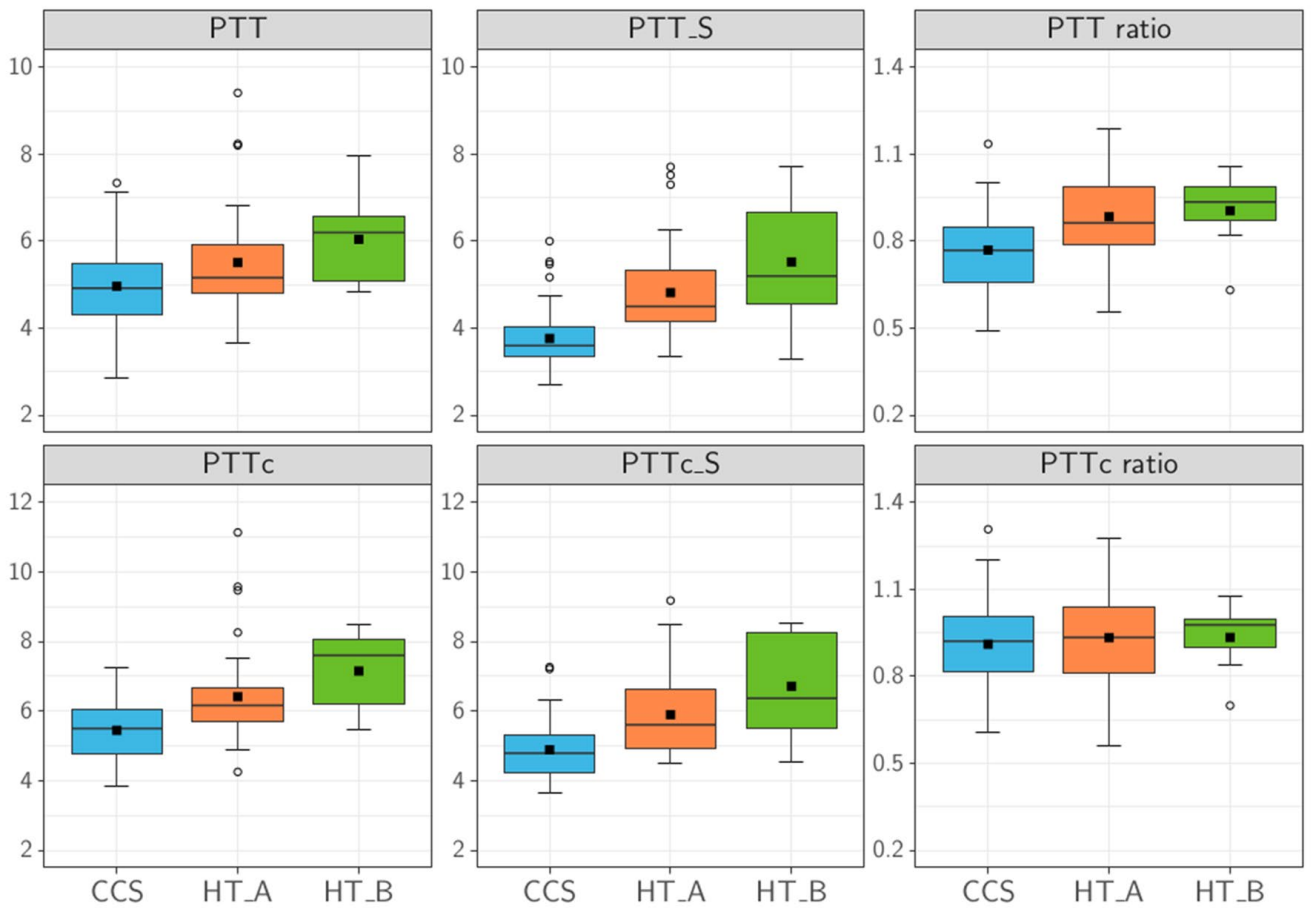
Pulmonary transit time parameters including stress and ratio biomarkers. The figure shows the comparison of pulmonary transit time (PTT) (s), stress pulmonary transit time (PTT_S) (s), pulmonary transit time ratio (PTT ratio) and heart rate corrected derived parameters (PTTc (s), PTTc_S (s) and PTTc ratio). Statistical analysis followed the corresponding methods section, α = 0.05 defined a statistically significant result. The average PTT in the childhood cancer survivors (CCS) group was not significantly lower than in the heart transplant without diastolic dysfunction subgroup (HTx_A) or heart transplant with diastolic dysfunction subgroup (HTx_B). PTTc, on the other hand, was significantly lower in both cases. Both PTT_S and PTTc_S were significantly lower in the CCS group than in the HTx subgroups. Stress transition times were shorter than rest times, and therefore ratio parameters were below 1 in all groups.

The complete results are summarized in Table 3. Boxplots for rest and stress transition times and derived ratio parameters are shown in Fig. 2.

**Table 3:** Pulmonary circulation biomarkers.

	CCS	HTx_A	HTx_B	HTx	p (CCS vs. HTx_A)	p (CCS vs. HTx_B)	p (HT_A vs. HTx_B)	p (CCS vs. HTx)
PTT (s)	4.96 ± 0.93	5.51 ± 1.14	6.04 ± 1.13	5.59 ± 1.14	0.063	0.13	0.61	**0.007**
PTTc (s)	5.45 ± 0.87	6.41 ± 1.3	7.15 ± 1.2	6.52 ± 1.30	** < 0.001**	**0.015**	0.44	** < 0.001**
PTB	5.82 ± 0.91	7.22 ± 1.62	8.43 ± 1.51	7.40 ± 1.65	** < 0.001**	** < 0.001**	0.38	** < 0.001**
PBVI (mL/m^2^)	238.98 ± 51.88	252.39 ± 64.32	245.26 ± 71.09	241.78 ± 64.60	0.58	0.98	0.97	0.32
PTT_S (s)	3.76 ± 0.78	4.82 ± 1.03	5.52 ± 1.56	4.92 ± 1.13	** < 0.001**	**0.003**	0.37	** < 0.001**
PTTc_S (s)	4.89 ± 0.89	5.9 ± 1.14	6.71 ± 1.63	6.02 ± 1.24	** < 0.001**	**0.006**	0.33	** < 0.001**
PTB_S	6.46 ± 1.07	6.85 ± 1.51	7.86 ± 2.04	7.00 ± 1.61	0.78	0.20	0.65	0.13
PTT ratio	0.77 ± 0.14	0.88 ± 0.14	0.91 ± 0.14	0.89 ± 0.14	**0.002**	0.18	0.96	** < 0.001**
PTTc ratio	0.91 ± 0.16	0.93 ± 0.16	0.93 ± 0.13	0.93 ± 0.15	0.80	0.96	< 0.99	0.51

Values are presented as mean ± SD unless otherwise indicated.

Statistically significant p values are in bold.

*PTT* pulmonary transit time, *PTTc* Bazett's formula corrected pulmonary transit time, *PTB* pulmonary transit beats, *PBVI* pulmonary blood volume index, *PTT_S* stress pulmonary transit time, *PTTc_S* Bazett's formula stress pulmonary transit time, *PTB_S* stress pulmonary transit beats, *PTT ratio* pulmonary transition time ratio, *PTTc ratio* Bazett's formula corrected pulmonary transition time ratio, *HTx* heart transplant group, *HTx_A* heart transplant without diastolic dysfunction subgroup, *HTx_B* heart transplant with diastolic dysfunction subgroup.

### Rest parameters

The average PTT in the CCS group was not significantly lower neither in the HTx_A (4.96 ± 0.93 s vs. 5.51 ± 1.14 s, p = 0.063) nor the HTx_B group (4.96 ± 0.93 s vs. 6.04 ± 1.13 s, p = 0.13). Although values were lower in the HTx_A subgroup than the HTx_B, the differences did not meet conventional levels of statistical significance.

PTTc, on the other hand, was significantly lower in both cases (5.45 ± 0.87 s (CCS) vs. 6.41 ± 1.3 s, p < 0.001 (HTx_A) and vs. 7.15 ± 1.2 s, p = 0.015 (HTx_B) and the same applies for PTB (5.82 ± 0.91 (CCS) vs. 7.22 ± 1.62, p < 0.001 (HTx_A) and vs. 8.43 ± 1.51, p < 0.001 (HTx_B)). The comparison between HTx_A and HTx_B shows lower values in the first group but without statistical significance. There were no differences in PBVI values.

When comparing CCS and the HTx group as whole, the results copy those of comparing CCS and HTx_A subgroup with 1 exception—PTT was significantly prolonged in the HTx group.

### Stress parameters

Both PTT_S and PTTc_S were significantly lower in the CCS group than in the HTx groups: PTT_S 3.76 ± 0.78 s (CCS) vs. 4.82 ± 1.03 s, p < 0.001 (HTx_A) and vs. 5.52 ± 1.56 s, p = 0.003 (HTx_B) and PTTc_S: 4.89 ± 0.89 s (CCS) vs. 5.9 ± 1.14, p < 0.001 (HTx_A) and vs. 6.71 ± 1.63, p = 0.006 (HTx_B).

PTB_S showed lower values in the CCS group, but without statistical significance (6.46 ± 1.07 (CCS) vs. 6.85 ± 1.51, p = 0.78 (HTx_A) and vs. 7.86 ± 2.04, p = 0.19 (HTx_B). Similarly to the rest parameters, the comparison between HTx_A and HTx_B shows lower parameters in the first group, but without statistical significance.

Comparison of CCS and HTx group shows the same results as in the HTx_A group, both PTT_S and PTTc_S were significantly lower in the CCS group, PTB_S showed lower values without statistical significance.

### Ratio parameters

The PTT ratio was significantly lower in the CCS than in the HTx_A subgroup (0.77 ± 0.14 vs. 0.88 ± 0.14, p = 0.002) and HTx group as well (0.77 ± 0.14 vs. 0.89 ± 0.14, p < 0.001). Lower parameters without statistical significances were shown when comparing the CCS and the HTx_B groups (0.77 ± 0.14 vs. 0.91 ± 0.14, p = 0.20). Although there were no significant differences between the groups in the case of the PTTc ratio, the PTT ratio and the PTTc ratio were below 1 in all groups, which means PTT_S times were shorter than the rest values.

Regarding the BMI, no statistical significance in any group or parameter was found with only 1 exception—a positive correlation of BMI with PTTc ratio in the CCS group (r = 0.49, p = 0.002). In addition, age did not correlate with PTT-derived parameters in any group (all r values < 0.2, all p values > 0.05).

## Discussion

The main goals of this study were to provide more evidence about pulmonary circulation parameters acquired by first-pass perfusion CMR, to investigate stress parameters in the HTx population as a potential marker of diastolic dysfunction and to compare rest and stress parameters and present ratio parameters as potential new biomarkers. The main finding was that stress pulmonary circulation times with and without Bazett’s formula correction were shorter than rest times contrary to the only published article including stress data^7^. Additionally, more data about pulmonary circulation parameters in new populations and ratio parameters were presented. Since ratio biomarkers are calculated as the ratio of stress and rest values, they describe the heart reaction to adenosine-induced pharmacological stress.

In previously published data^7^, only HR corrected PTT time was assessed from stress biomarkers and it showed prolongation or no change compared to HR corrected PTT rest time. In the whole study population of 69 HCM patients, the results showed overall prolongation of PTT stress time. When divided into subgroups based on left atrial pressure (LAP), the corrected PTT stress was prolonged in patients with normal LAP, and in increased LAP, the values stayed roughly the same. Therefore, the calculated corrected PTT ratio would have been higher than 1 or close to 1 for this population.

In this study, all presented groups had PTT and PTTc ratios lower than 1, and therefore stress biomarkers were shorter, which is in contrary. One explanation could be based on the diastolic dysfunction in the HCM group. However, this difference is certainly a matter of discussion. Although adenosine indeed induces vasodilation in pulmonary circulation as the authors claim^7^, its overall effect on the myocardium and circulation itself is still debatable and more complex. The HR increase is well documented^20,21^, and our data are no exception. However, its effect on contractility is quite controversial. Some studies showed a positive outcome on contractility^22^ or stated it affects beta-receptors as well^23^, while others claim it has an indirect effect on contractility but no beta-receptor activity^24^. Apart from a direct reaction on the myocardium, recent reports focused on its repercussions in the central nervous system (CNS), mainly in the *nucleus tractus solitarius* but other parts of CNS as well^25^, and its possible influence on baroreflex via CNS^26^. However, most of the studies were on rats or mice models or isolated myocardial cells, and for example, the effect on baroreflex could be indirect via hypotension from vasodilatation resulting in increasing HR and contractility of the myocardium. This truly complex problem is quite beyond the scope of this paper to discuss in all the possibilities.

Overall, we presume that in a healthy population, stress biomarkers such as PTT_S should be shorter than their rest analogues, therefore, ratio biomarkers should be less than 1, and they could be potentially used as additional markers of the myocardial reserve and/or diastolic dysfunction. However, further research, including a comprehensive healthy population, is needed.

We hypothesized underlying diastolic function impairment in patients after HTx and the feasibilty of using pulmonary circulation and potentially ratio parameters to assess it. During diastole, the LV receives blood from the left atrium (LA). LV diastolic dysfunction leads to the impairment of its filling function, therefore filling pressure in the LV is increased, which leads to increased pressure in the LA as well. This corresponds with increasing of the pulmonary capillary wedge pressure (PCWP). When PCWP is increased, the whole process of blood circulating through the pulmonary circulation is supposedly impaired as well, resulting in longer PTT times. This mechanism is supposedly more appreciable in stress conditions, which agrees with our results and also possibly explains the difference in stress CO between CCS and HTx_A.

As Meluzin et al. reported^10^—16% of HTx subjects had signs of diastolic dysfunction in rest conditions (14.6% in this study), but other 58% showed signs of diastolic impairment in stress conditions^10^. While the difference in PTT was not significant, PTTc, PTT_S and PTTc_S were significantly lower in the CCS group than the HTx_A subgroup, which follows our assumption. In addition, both rest and stress transit times reported by Ricci et al. in HCM patients were higher^7^. Since LV hypertrophy in HCM patients is most commonly paired with diastolic dysfunction, these results further strengthenour assumptions.

The lower PTT ratio, while constant PTTc ratio between groups, could be influenced by the difference in HR and the results could be affected by the heart denervation in the HTx population, but that would not explain the tendency to higher values in the HTx_B group with diastolic dysfunction as to the HTx_A. Combined with the prolongation of stress parameters, and thus ratio parameters around 1 or more, as reported by Ricci et al. in HCM patients^7^, this further-more points to diastolic function impairment as the explanation.

However, additional studies with different populations are necessary to confirm these results.

In all cases, no statistically significant differences were found when comparing the HTx_A and the HTx_B groups. However, in cases such as for the PTTc (6.41 ± 1.3 s vs. 7.15 ± 1.2 s, p = 0.44) or PTTc_S (5.9 ± 1.14 s vs. 6.71 ± 1.63 s, p = 0.33), a tendency towards lower values in HTx_A was observed. This is limited by the number of patients included in the HTx_B group. Although this number of patients with diastolic dysfunction included was relatively small, impacting the implications of the results, the proposed method illustrates the feasibility of including pulmonary circulation biomarkers as an additional method to assess diastolic dysfunction.

Since the limited number of subjects in HTx_B subgroup, HTx group as whole was included and compared with CCS group—the results resembled the comparison of CCS and HTx_A group with the excemption of the PTT, which was significantly longer in the HTx group. This result further documents an assumption of the influence of an underlying dysfunction.

It is not completely clear whether to prefer HR-corrected PTT times or not. In Ricci’s study, both rest times and only corrected stress PTT times were reported^7^, and in Houard et al.^6^ no HR correction was applied at all. We believe both alternatives are justified. While PTB could be a form of overriding the effect of HR in counting the number of cycles, for the stress parameters and ratios, we think that both possibilities are valid and should be acquired.

It is also possible to evaluate these biomarkers from the peak-to-peak time interval in the signal intensity curve. However, we opted for the onset assessment because the first appearance of the contrast showed good reproducibility.

To our knowledge, only one other study acquiring pulmonary circulation stress biomarkers was published to date, and therefore, with 87 patients included, this is one of the first larger CMR-based studies involving stress circulation parameters. However, more studies have to be made to confirm our results, including different groups with reduced LVEF, other diagnoses, and an aged-match healthy control group, although applying adenosine to a healthy control group could prove ethically problematic. On the other hand, when using the presented or an earlier developed method, a center with a relatively high number of stress perfusions could comfortably conduct other studies retrospectively to gather more data about stress circulation biomarkers. As far as we know, these parameters have never been studied before in CCS or HTx patient groups.

### Study limitations

We acknowledge there are limitations to this study. It was a single-center retrospective study, but arguably the principal constrain is the participant’s selection: CCS and HTx. CCS cannot be reffered to as a healthy control group. Although in thorough examinations no signs of systolic or diastolic function impairment, other pathological results or any other cardiac function impairment were observed, patients included underwent potentially cardiotoxic chemotherapy. Their comparability is further limited by basic clinical parameters, mainly age and BMI. However, in neither group the age correlated with pulmonary circulation biomarkers, and the BMI correlated only with the PTTc ratio in the CCS group. Also, selecting patients after HTx could lead to bias with the adenosine effect on a denervated heart. Data on using adenosine or regadenoson on subjects after HTx is limited. In older studies, a hypersensitivity was described^27^, but it seems safe to administer it^28^. A newer study from Spain showed its use in the case of stress perfusion as well, although HTx patients presented a lower myocardial perfusion reserve index in comparison to healthy controls^29^. Furthermore, although the number of participants with diastolic dysfunction (HTx_B group) was low, impacting the implications of the results, the proposed method illustrates the feasibility of including pulmonary circulation biomarkers as a potential additional approach to assess diastolic dysfunction.

### Ethics approval and consent to participate

This retrospective study was performed in accordance with the Declaration of Helsinki (2000) of the World Medical Association. The Ethics Committee of the Faculty of Medicine, Masaryk University, confrmed under reference number 02082021, that this study has been performed on data of patients already participating in other research studies with signed Informed Consent about using their data anonymously for research purposes and therefore according to the Czech legislation, no specific new approval of the ethics committee was required. Only participants who signed written Informed Consent about using their data anonymously for research purposes were enrolled in the study. No patient from potentially vulnerable group was enrolled in the study.

## Conclusions

The study provided more evidence about pulmonary circulation parameters acquired by first-pass perfusion CMR, investigated stress parameters in an HTx population as a potential marker of diastolic dysfunction and presented ratio parameters as potential new biomarkers.

The results showed lower values of PTTc, PTT_S and PTTc_S in the CCS group, corresponding with the presumption of underlying diastolic dysfunction in HTx patients. As assumed, contrary to the data published for HCM patients^7^, both PTT_S and PTTc_S times were shorter than their rest analogues in all groups, and therefore the PTT ratio and the PTTc ratio were less than 1.

## Data Availability

The datasets analysed during the current study are available from the corresponding author upon reasonable request.

## Abbreviations

BMI: Body mass index
b-TFE: Balanced turbo field echo
CCS: Childhood cancer survivors
CMR: Cardiovascular magnetic resonance
CNS: Central nervous system
CO: Cardiac output
DD: Diastolic diameter
DD LV: Left ventricular diastolic diameter
HCM: Hypertrophic cardiomyopathy
HR: Heart rate
HTx: Heart transplant
HTx_A: Heart transplant without diastolic dysfunction subgroup
HTx_B: Heart transplant with diastolic dysfunction subgroup
IR-TFE: Inversion-recovery turbo field echo
LA: Left atrium
LAP: Left atrial pressure
LGE: Late gadolinium enhancement
LV: Left ventricle
LVEF: Left ventricular ejection fraction
LV LGE: Left ventricular late gadolinium enhancement
LVOT: Left ventricular outflow tract
LV SV: Left ventricular stroke volume
MRI: Magnetic resonance imaging
PBVI: Pulmonary blood volume index
PCWP: Pulmonary capillary wedge pressure
PH: Pulmonary hypertension
PSIR: Phase-sensitive inversion recovery
PTB: Pulmonary transit beats
PTB_S: Stress pulmonary transit beats
PTT: Pulmonary transit time
PTTc: Bazett's formula corrected pulmonary transit time
PTT_S: Stress pulmonary transit time
PTTc_S: Bazett's formula corrected stress pulmonary transit time
PTT ratio: Pulmonary transit time ratio
PTTc ratio: Bazett's formula corrected pulmonary transit time ratio
ROIs: Regions of interest
RV: Right ventricle
RV SV: Right ventricular systolic volume
SAX: Short-axis
SI: Signal intensity
sPAP: Systolic pulmonary artery pressure
SSFP: Steady-state free precession
TE: Echo time
TR: Repetition time
TTE: Transthoracic echocardiography
